# Benzocaine-Induced Cyanosis

**Published:** 2016-05-16

**Authors:** Ankit Jain, Anuradha Patel, Ian C. Hoppe

**Affiliations:** ^a^Department of Anesthesia, Rutgers New Jersey Medical School, Newark, NJ; ^b^Department of Plastic Surgery, Rutgers New Jersey Medical School, Newark, NJ

**Keywords:** Benzocaine, Methemoglobinemia, Cyanosis, Desaturation, Methylene Blue

## DESCRIPTION

**Figure 1:**
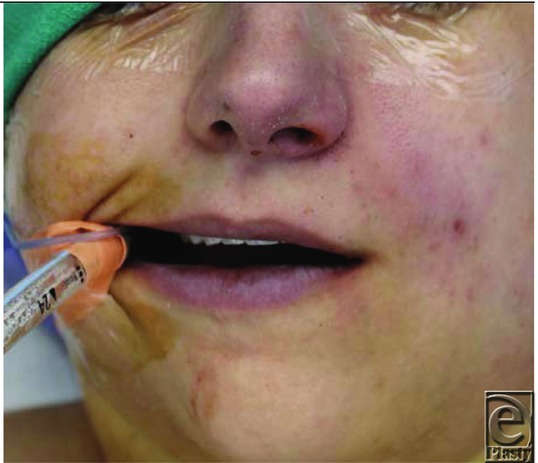
Cyanosis.

**Figure 2:**
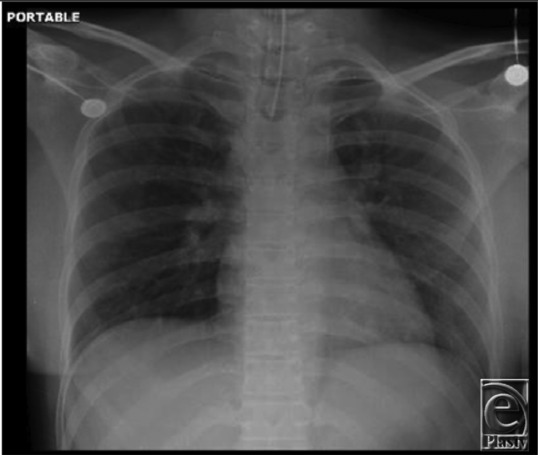
A chest x-ray showing correct endotracheal tube placement and no acute lung pathology.

A 28-year-old woman underwent fiberoptic intubation for incision and drainage of joint space infection of a temporomandibular joint arthroplasty performed 10 days prior. The airway was prepared with 4 sprays of topical benzocaine spray and 3% lidocaine ointment. After 30 minutes, the peripheral oxygen saturation (Spo_2_) dropped gradually to 85% and the patient's lips and face showed evidence of cyanosis (Fig 1). The finding from a chest x-ray film was negative for any acute lung pathology and the endotracheal tube was noted to be above the carina (Fig 2).

## QUESTIONS

1. **What is the most likely diagnosis in this case and how does it affect oxygenation?**
2. **What are the predisposing factors for development of this condition?**
3. **What are the clinical features of this condition?**
4. **How will you diagnose and treat this condition?**

## DISCUSSION

**Figure 3:**
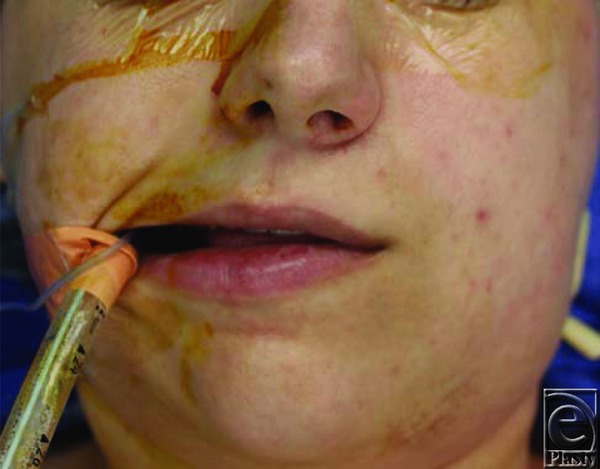
The appearance following the administration of methylene blue showing resolution of cyanosis.

In this patient, arterial blood gas analysis showed a Pao_2_ of 546 mm Hg on 100% Fio_2_, and color of blood was noted to be chocolate brown. “CO-oximetric” blood gas analysis showed methemoglobin level of 29.3 g%. Treatment with 1 mg/kg of 1% methylene blue intravenously resulted in improvement of Spo_2_ to 99% within 15 minutes and repeat blood gas analysis showed methemoglobin level at 5.9 g%. Her clinical appearance improved as well with resolution of cyanosis (see later). She was extubated uneventfully when serial blood gas analysis showed a decreasing trend of methemoglobin levels. She was alert, oriented and with no evidence of any cognitive dysfunction and was discharged home the next day. Methemoglobin is formed when the divalent iron molecule of the normal hemoglobin is converted to a trivalent state.^1^^-^3 Normally, 1% to 2% of methemoglobin is present physiologically. Methemoglobin is continuously formed in red blood cells and is reduced to deoxyhemoglobin by nicotinamide adenine dinucleotide phosphate (NADPH) methemoglobin reductase enzyme.^4^ Methemoglobinemia is a condition that results from elevated levels of methemoglobin. Elevated levels of methemoglobin give a characteristic chocolate color to the blood. Cyanosis unresponsive to oxygen with evidence of “dark chocolate” colored blood is very suggestive of methemoglobinemia.^5^ Methemoglobin is unable to bind and carry oxygen because of its trivalent ferric state. High levels of methemoglobin disrupt oxygenation by two mechanisms. First, methemoglobin reduces the oxygen-carrying capacity of blood; second, the presence of oxidized iron changes the heme tetramer in a way that reduces oxygen release in the tissues, thus shifting the oxyhemoglobin dissociation curve to the left, as in alkalosis. Thus, it creates a state of “functional anemia” (see Fig 3).

There are multiple predisposing factors for development of methemoglobinemia, namely drugs like local anesthetics, antimalarials (chloroquine, primaquine), nitrites or nitrates, inhaled nitric oxide, nitroprusside, sulfonamides, metoclopramide, and phenytoin. These drugs inhibit the NADPH enzyme directly or indirectly. Other factors include a break in the mucosal barrier, excessive use of a topical agent, and partial or complete deficiency of methemoglobin reductase enzyme.^6^ We believe that the break in the mucosal barrier as a result of recent oral surgery played a major role in the development of cyanosis and methemoglobinemia in our patient.

The clinical features of methemoglobinemia are similar to signs and symptoms of hypoxia. The methemoglobin level determines the presenting symptoms. Normally, 5 g/dL of methemoglobin produces cyanosis, but as little as 1.5 g/dL can produce noticeable cyanosis. Clinical symptoms are lacking with methemoglobin levels lower than 10%. Concentrations of 10% to 15% produce visible cyanosis unresponsive to oxygen therapy, and blood may appear burgundy-brown in color. Concentrations greater than 20% result in symptoms related to tissue hypoxia and include anxiety, fatigue, dyspnea, lethargy, tachycardia, headache, and syncope. Levels more than 50% can result in stupor and coma, and death has been reported with levels higher than 70%.^4^

Diagnosis of methemoglobinemia starts with clinical findings. Cyanosis unresponsive to increasing concentration of oxygen along with the use of an agent known to cause methemoglobinemia should raise suspicion about this condition. A normal arterial blood gas analysis with a pulse oximetry saturation around 85% should prompt a “co-oximetric” analysis to rule out methemoglobinemia. Normal blood gas analysis measures dissolved oxygen, which under normal conditions is in equilibrium with oxygen carried by hemoglobin. Therefore, a blood gas analysis may show Pao_2_ and saturation within normal range despite impaired oxygen-carrying capacity. Treatment of this condition is achieved mainly by administering 1 to 2 mg/kg of 1% methylene blue intravenously. Methylene blue acts as a reducing agent via the NADPH methemoglobin reductase pathway. Methylene blue is reduced in red blood cells, and presumably in tissues, to leucomethylene blue. Leucomethylene blue reacts spontaneously with high concentrations of methemoglobin in blood, rapidly reducing methemoglobin to hemoglobin, even in the presence of oxygen. Leucomethylene blue also converts ferric iron back to the ferrous state and restores the oxygen-carrying capacity of hemoglobin. Cyanosis usually resolves within 15 to 30 minutes. Marked reduction in methemoglobinemia, usually by 50%, is seen within 30 to 60 minutes.^7^ A repeat dose of 1 mg/kg can be given within 1 hour if cyanosis and symptoms do not subside. In severe cases, hyperbaric oxygen therapy and exchange transfusion can be used when patients do not respond to methylene blue.^8^ Methylene blue itself can be toxic and not free from side effects. Common side effects include but are not limited to hypertension, precordial pain, dizziness, confusion, nausea, vomiting, discoloration of urine, and anemia. Methylene blue is a monoamine oxidase inhibitor and, if infused intravenously at doses exceeding 5 mg/kg, may precipitate serious serotonin toxicity and serotonin syndrome. It causes hemolytic reactions in carriers of the glucose-6-phosphate-dehydrogenase enzymatic deficiency.

## SUMMARY

Unexplained cyanosis in the absence of other hemodynamic disruptions, in a patient with a history of use of benzocaine spray, should alert the physician for methemoglobinemia. The diagnosis rests mainly on clinical features like cyanosis, “chocolate colored” blood, cyanosis unresponsive to oxygen therapy, and a CO oximetric analysis of elevated methemoglobin level. Methylene blue resolves the symptoms in most cases and should be readily available in any area where benzocaine is frequently used. Physicians should avoid the use of topical anesthetics spray in inflamed or denuded mucosal surfaces as the rate and the amount of absorption is unpredictable.
